# Role of prophylactic cranial irradiation in patients with limited disease small cell lung cancer: A Danish single institution cohort

**DOI:** 10.2340/1651-226X.2025.43935

**Published:** 2025-07-25

**Authors:** Sara Linde, Marianne Maquard Knap, Lone Hoffmann, Azza Ahmed Kahlil, Christina Maria Lutz, Maria Kandi, Lise Saksø Mortensen, Ditte Sloth Møller, Hjørdis Hjalting Schmidt

**Affiliations:** a Department of Oncology, Aarhus University Hospital, Aarhus, Denmark; b Department of Clinical Medicine, Aarhus University, Aarhus, Denmark; c Department of Oncology, Gødstrup Regional Hospital, Gødstrup, Denmark

**Keywords:** Prophylactic cranial irradiation, small cell lung cancer, limited disease, overall survival, cohort

## Abstract

**Background and purpose:**

Prophylactic cranial irradiation (PCI) is part of standard treatment for patients with limited disease small cell lung cancer (LD-SCLC), treated with curative intent. However, doubt has been raised about the efficacy of PCI in a modern clinical setting. Therefore, we examined factors impacting PCI receival, the cumulative incidence of symptomatic brain metastases, and overall survival (OS) with and without PCI.

**Patient/material and methods:**

Records of 190 patients with LD-SCLC consecutively treated between 2012 and 2021 at our institution were reviewed. Patients were grouped based on whether they received PCI (PCI, *n* = 119) or not (no PCI, *n* = 71). Baseline characteristics, Kaplan-Meier estimates of OS, and cumulative incidence of symptomatic brain metastases were compared for the two groups.

**Results:**

PCI no patients were older, had a poorer performance status, were more often treated in 2018–2021 and had more frequently a brain magnetic resonance imaging (MRI) at the time of diagnosis. No PCI median OS was 19 months compared to 24 months for PCI, not significantly different (*p* = 0.40). During follow-up 54 patients (28.4%) developed symptomatic brain metastases, with no statistically significant difference in the numbers of patients with, and cumulative incidence of, symptomatic brain metastases between the two groups (*p* = 0.35 and *p* = 0.21, respectively).

**Interpretation:**

Despite patients not receiving PCI being older and in poorer performance status, no statistically significant difference in OS or cumulative incidence of brain metastasis were observed compared to patients who received PCI. This supports uncertainty regarding the role of PCI.

## Introduction

Prophylactic cranial irradiation (PCI) with 25Gy in 10 fractions (fx), 5fx per week, following concurrent chemo-radiotherapy, is the standard treatment for patients with limited disease small cell lung cancer (LD-SCLC), with responsive disease [1, 2]. Despite being treated with curative intent, the prognosis is poor, with a 5-year survival rate of less than 30% and a high risk of both local and distant failure [2–6]. Brain metastasis is a significant concern, with nearly half of patients developing symptomatic brain metastasis within the first 2 years after treatment [7]. A landmark meta-analysis from 1999 demonstrated a 5% overall survival (OS) benefit and a reduced incidence of brain metastasis in patients with LD-SCLC treated with PCI [8]. The use of PCI was further supported in 2007 by a randomized trial finding a decreased risk of symptomatic brain metastasis and better OS in patients with extensive disease small cell lung cancer (ED-SCLC) [9]. However, most patients did not have sufficient brain imaging to rule out brain metastases before initiating PCI. Based on brain magnetic resonance imaging (MRI), up to 15% of patients with small cell lung cancer (SCLC) have asymptomatic brain metastases at the time of PCI [10, 11]. Modern diagnostic workup is more extensive, including combined positron emission tomography (PET) and computed tomography (CT), and a brain MRI [1, 5]. A meta-analysis from 2024 found no OS benefit for patients with LD-SCLC or ED-SCLC receiving PCI following a brain MRI confirming the absence of brain metastases [12]. A randomized trial of a surveillance program with brain MRI versus PCI and brain MRI in patients with ED-SCLC from 2017 showed no survival benefit of PCI, but a significantly lower number of brain metastases with an incidence of 15% versus 46% at 6 months, 33% versus 59% at 12 months and 40% versus 64% at 18 months [13]. A limitation of PCI is the risk of neurocognitive side effects; approximately 83% of patients over 60 years of age have chronic neurocognitive impairment 12 months after PCI, compared with only 56% of patients younger than 60 years of age [14]. Hippocampal avoidance in PCI has been investigated in several trials as a strategy to reduce the neurocognitive impairment, the results are conflicting [15–17]. International guidelines recommend PCI for all fit patients with LD-SCLC. For patients not surely fit for PCI, shared decision-making is recommended [1, 18]. The conflicting results on effect and side effects of PCI make the shared decision-making process complex and not well supported. In recent randomized studies, the proportion of patients receiving PCI varies from 54% to 84% [4, 5, 19, 20]. This study aims to examine the role of PCI on OS and symptomatic intracranial failure in a historical Danish cohort of 190 patients diagnosed and treated by modern standards, as well as investigating factors influencing the selection of patients who receive PCI.

## Patients/material and methods

### Patients

All patients with LD-SCLC treated at Aarhus University Hospital between 2012 and 2021 with curatively intended thoracic radiotherapy (45Gy/30fx/10fx per week) were identified from radiotherapy records and patient data were reviewed. A physician manually extracted data from hospital records, radiotherapy plans, and imaging. No data were missing.

Pre-therapeutic staging included diagnostic 18 fluoro-deoxyglucose PET (^18^F-FDG-PET), chest and abdominal CT and endobronchial ultrasonography (EBUS). From 2017, brain MRI became part of diagnostic workup. Diagnosis was confirmed by biopsy of tumors and/or lymph nodes. Patients received 0–4 cycles of platinum-based chemotherapy. Carboplatin (AUC 5, according to the Calvert formula) or cisplatin (75 mg/m²) were administered on day 1 of each cycle together with etoposide on days 1 to 3 (120 mg/m² intravenously or 240 mg/m² orally). An ^18^F-FDG-PET and a 4D-CT were obtained shortly before radiotherapy treatment started and used for treatment planning. Radiotherapy was delivered as homogeneous doses of 1.5Gy per fraction, twice daily, with daily treatment setup imaging (Cone-beam CT). All patients received intensity-modulated radiation therapy (IMRT) and from 2013 soft tissue match with adaptive radiotherapy was used for setup [21]. After chemo-radiotherapy patients without progressive disease and in good clinical condition were offered PCI (25Gy/10fx/5fx per week) at physicians’ discretion. The cohort was divided into two groups: those who received PCI (PCI) and those who did not (no PCI). Follow-up consisted of a contrast-enhanced thoracic and abdominal CT-scan every 3 months until 2 years, hereafter every 6 months until 5 years. Systematic brain imaging was not routinely performed during follow-up. If relapse was suspected, patients underwent re-evaluation and restaging using ^18^F-FDG-PET, CT scans, and biopsies. A brain MRI or CT was performed in case of symptoms consistent with brain metastasis. ECOG performance status (PS) was grouped as 0–1 or 2–3; stage as IB-IIB, IIIA-IIIB, or IIIC; and treatment period as early (from 1^st^ of January 2012 to 31^st^ of December 2017) or late (from 1^st^ of January 2018 to 31^st^ of December 2021). Symptomatic brain metastasis was defined as any documented event during follow-up.

### Statistics

Statistical analyses were performed in SPSS 28.0. and R 4.4.1. Comparisons of baseline characteristics were made by Chi-square (χ^2^) test, except for age where an independent samples T-test was used. Age was normally distributed, assessed by QQ-plot. Parameters that significantly impacted receival of PCI were defined as *p* < 0.05. Follow-up time was estimated by inverse Kaplan-Meier. OS was defined as time from the start of radiotherapy to death. Time to symptomatic brain metastasis was defined as time from start of radiotherapy to first CT or MRI scan of the brain confirming brain involvement. Kaplan-Meier estimates of OS were compared in groups using Cox Regression, with significance reached if *p* < 0.05. The univariate Cox Regression was performed for sex, PS, stage, chemotherapy agent, treatment period, and PCI on OS. Cumulative incidence curves were used to estimate the cumulative risk of symptomatic brain metastases grouped by PCI. Gray’s test was used to compare the groups, with death without symptomatic brain metastases as a competing risk. Finally, a multivariate Cox Regression analysis on sex, PS, stage, treatment period, and PCI was performed to assess independent predictors of OS.

## Results

**Figure 1:**
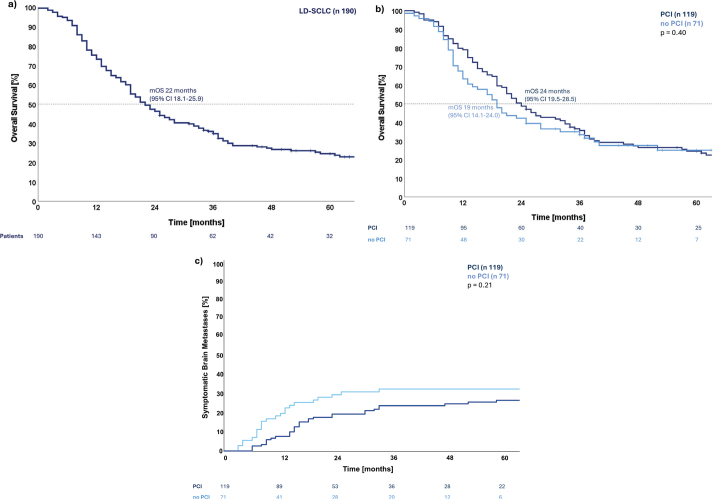
(a) Kaplan-Meier curve for overall survival for the cohort of 190 patients with limited disease small cell lung cancer treated at a single institution in Denmark between 2012 and 2021 with a curative intent. (b) Kaplan-Meier curves for overall survival grouped for patients receiving prophylactic cranial irradiation or not. Compared with Cox-regression *p* = 0.40. (c) Cumulative incidence of symptomatic brain metastases grouped for patients receiving prophylactic cranial irradiation or not. Compared with Gray’s test with death as a competing risk *p* = 0.21.

A total of 190 patients with LD-SCLC were registered in the radiotherapy records with curatively intended thoracic radiotherapy of 45Gy/30fx/10 fx per week, from January 2012 to December 2021. Data extraction was performed on the 26^th^ of November 2023. A total of 119 patients (62.6%) received PCI (PCI group) and 71 patients (37.4%) did not (no PCI group). There was no difference between the two groups in terms of sex, disease stage, smoking status, or chemotherapy regimen. No PCI patients were older, with a median of 70.8 years [43.5–83.1] compared to PCI patients with 64.5 years [40.7–80.2] (*p* < 0.01) and had a worse PS (*p* = 0.05). Fewer no PCI patients recieved cisplatin than PCI patients (*p* < 0.01) and they were more likely to have been treated in the later period (2018–2021) (*p* < 0.01) and to have undergone brain MRI at diagnosis (*p* < 0.01), see Table 1. The frequency of PCI decreased from 82.5% in the early treatment period to 36.5% in the late period, while the frequency of diagnostic brain MRI increased from 26.9% to 89.0% in the same periods. After a median follow-up time of 74 months, the median OS (mOS) for the entire cohort was 22 months (95% CI 18.1–25.9), with a 2-year survival of 46.3 % (95% CI 39.2–53.4) and a 5-year survival of 24.5 % (95% CI 18.0–31.0), see Figure 1a. The mOS was 19 months (95% CI 14.1–24.0) for no PCI patients and 24 months (95% CI 19.5–28.5) for PCI patients, with no significant OS difference (*p* = 0.40), see Figure 1b.

**Table 1:** Baseline characteristics of 190 patients.

Characteristics	no PCI *N* = 71	PCI *N* = 119	*p*
**Age**, years (median) [min-max]	70.8 [43.5–83.1]	64.5 [40.7–80.2]	<0.001*
**Sex**			0.525
Male	36 (50.7%)	66 (55.5%)	
Female	35 (49.3%)	53 (44.5%)	
**ECOG PS**			**0.049**
0–1	55 (77.5%)	105 (88.2%)	
2–3	16 (22.5%)	14 (11.8%)	
**Smoking status**			0.426
Current	40 (56.3%)	74 (62.2%)	
Former	31 (43.7%)	45 (37.8%)	
**Stage**			0.116
IB–IIB	18 (25.4%)	16 (13.4%)	
IIIA–IIIB	40 (56.3%)	77 (64.7%)	
IIIC	13 (18.3%)	26 (22.0%)	
**Diagnostic MRI of the brain**	53 (74.6%)	49 (41.2%)	**<0.001**
**Chemotherapy agent**			**<0.001**
Cisplatin and Etoposide	10 (14.1%)	54 (45.4%)	
Carboplatin and Etoposide	56 (78.9%)	64 (53.8%)	
Etoposide only or no chemotherapy	5 (7.0%)	1 (0.8%)	
**Chemotherapy regimen**			0.113
Concurrent	62 (87.3%)	106 (89.1%)	
Sequential	5 (7.0%)	12 (10.1%)	
No chemotherapy	4 (5.6%)	1 (0.8%)	
**Treatment period**			**<0.001**
Early (2012–2017)	19 (26.8%)	89 (74.8%)	
Late (2018–2021)	52 (73.2%)	30 (25.2%)	

PCI: prophylactic cranial irradiation; ECOG PS: ECOG PS: Eastern Cooperative Oncology Group Performance Status; MRI: magnetic resonance imaging.

*P*’s are calculated with Chi-square (χ^2^) test except * which is calculated with independent samples *T*-test.

Significance reached if p < 0.05, this is maked in bold.

In the univariate analysis, stage was the only variable significantly associated with OS. This association remained significant in the multivariate analysis, which included sex, PS, stage, chemotherapy agent, treatment period, and PCI, see Table 2 and the supplementary material.

**Table 2:** Univariate and multivariate Cox regression for Overall Survival.

Variable	Univariate Cox regression		Multivariate Cox regression	
	HR* (95% CI)	*p*	HR* (95% CI)	*p*
**Sex**				
Female	Ref.		Ref.	
Male	0.89 (0.64–1.22)	0.46	0.99 (0.71–1.38)	0.94
**ECOG PS**				
0–1	Ref.		Ref.	
2–3	1.19 (0.77–1.85)	0.43	1.25 (0.78–1.99)	0.36
**Stage**				
IB–IIB	Ref.		Ref.	
IIIA–IIIB	1.62 (1.01–2.58)	**0.04**	1.65 (1.03–2.64)	**0.04**
IIIC	2.40 (1.40–4.10)	**<0.01**	2.57 (1.48–4.46)	**<0.01**
**Chemotherapy agent**				
Carboplatin/Etoposide, Etoposide or none	Ref.		Ref.	
Cisplatin/Etoposide	0.99 (0.71–1.39)	0.96	1.11 (0.76–1.62)	0.61
**Treatment period**				
Early (2012–2017)	Ref.		Ref.	
Late (2018–2021)	0.90 (0.62–1.26)	0.53	0.77 (0.52–1.15)	0.21
**PCI**				
No	Ref.		Ref.	
Yes	0.87 (0.62–1.21)	0.40	0.70 (0.47–1.05)	0.08

CI: confidence interval; PCI: prophylactic cranial irradiation; ECOG PS: Eastern Cooperative Oncology Group Performance Status

* Hazard Ratio (HR) for death.

Significance reached if p < 0.05, this is maked in bold

During follow-up, 54 patients (28.4%) developed symptomatic brain metastases: 23 events (32.4%) in the no PCI group and 31 (26.1%) in the PCI group. Fifteen events were first-time relapses without other signs of local or distant recurrences, with 8 (11.3%) in the no PCI group and 7 (5.9%) in the PCI group. The cumulative incidence of symptomatic brain metastases did not differ significantly between the two groups, with a hazard ratio of 0.71 (95% CI 0.41–1.21) (*p* = 0.21); however, visual inspection suggests that symptomatic brain metastases occurred earlier in the no PCI patients compared to the PCI patients. Demonstrating a 1-year risk of symptomatic brain metastases of 19.7% (95% CI 10.4–29.0) for no PCI and 7.6% (95% CI 2.8–12.3) for PCI and a 2-year risk of 29.6% (95% CI 18.9–40.3) and 19.3% (95% CI 12.2–26.5), respectively See Figure 1c.

## Discussion and conclusion

This study found a decreasing proportion of patients treated with PCI over time. Patients who did not receive PCI were older, had poorer PS, underwent brain MRI at diagnosis more frequently, and were more often treated during the later period of the study. Despite these differences, no significant differences in OS or cumulative incidence of symptomatic brain metastases were observed between the groups. A limitation of our study is that the single institution cohort may not have been large enough to detect a survival benefit, particularly since no individual study in the Auperin et al. (1999) meta-analysis had a significant OS benefit on its own [8]. The use of brain MRI largely increased over the study period and is now recommended as part of the diagnostic workup [1]. A brain MRI detects brain metastasis in nearly 25% of patients with SCLC, 11% of these being asymptomatic [11]. The survival benefit of PCI observed in previous studies may, in part, be explained by the fact that PCI was not solely prophylactic [8, 9]. This is supported by Gaebe et al. (2024), finding no significant OS difference between PCI or not in patients who underwent a brain MRI to exclude brain metastases, with a hazard ratio of 0.74 (95% CI 0.52–1.05) [12].Brain MRI at diagnosis likely contributed to the decrease in the proportion of patients treated with PCI in the later period due to the more comprehensive information provided by MRI, on potential frailty and increased risk of neurocognitive impairment (such as chronic ischemia, prior infarcts, and cerebral atrophy) [22–24]. Another contributing factor may have been the study by Takahashi et al., which found no survival benefit of PCI [13]. However, in our study regular MRI surveillance was not performed, and to omit PCI on the basis of the Takahashi et al. study, might be an over-interpretation, considering several patients in the surveillance group of that study received therapeutic radiotherapy to the brain afterward [13]. Large, randomized trials in LD-SCLC have also not implemented regular MRI surveillance of the brain in the follow-up, indicating it is not standard procedure [4, 5, 20]. The lack of systematic brain imaging in our study is a limitation; the number of brain metastases would likely have been larger, and there might have been a significant difference in the cumulative incidence of brain metastases. Development of brain metastases is often associated with severe symptoms that are difficult to palliate and death often follows shortly after [25, 26]. Data comparing neurocognitive side effects of PCI and symptoms of brain metastases are sparse. Takahashi et al. found no significant difference in neurocognitive function measured by the Mini-Mental State Examination between patients receiving PCI or not [13]. Ongoing randomized phase III studies, the MAVERICK trial (NCT04155034) and the PRIMALung trial (NCT04790253), are comparing PCI and MRI surveillance versus MRI surveillance alone for patients with SCLC. These trials include both LD-SCLC and ED-SCLC, with stratification for disease stage. Both trials have secondary endpoints examining cognitive failure; additionally, MAVERICK is examining toxicities, while PRIMALung is following patients with quality-of-life assessments. These trials will further examine the role of PCI in a modern era with immunotherapy emerging in the treatment of patients with LD-SCLC, following the ADRIATIC trial finding a survival benefit of durvalumab over placebo with a mOS of 55.9 months (95% CI 37.3-not reached) for durvalumab compared to 33.4 months (95% CI 25.5–39.9) for placebo [20]. Even though stratification for PCI was performed in the trial, the study was not powered to examine subgroup comparisons. Immunotherapy has been used for patients with ED-SCLC for some time, but data on PCI integrated with immunotherapy treatment is still sparse [1, 27, 28]. However, a retrospective study of ED-SCLC found a survival benefit with a hazard ratio of 0.72 (95% CI 0.58–0.88) for those receiving PCI after chemoimmunotherapy versus those only receiving chemoimmunotherapy [29]. The patients in our study were treated before immunotherapy was introduced, but the role of PCI for future patients treated with immunotherapy is uncertain regarding toxicities and efficacy.

In conclusion, despite PCI being offered to patients with favorable prognostic factors, this analysis of 190 patients showed no significant improvement in OS or cumulative incidence of symptomatic brain metastases for patients treated with PCI at a single institution. In the modern era of more extensive diagnostic workup and with immunotherapy emerging, the role of PCI seems uncertain. The data provided in this study support this uncertainty.

## Supplementary Material



## Data Availability

This study is based on healthcare data and cannot be publicly shared due to patient confidentiality and legal restrictions. An anonymized dataset may be made available upon reasonable request and subject to institutional approval.

## References

[1] Dingemans AMC, Früh M, Ardizzoni A, Besse B, Faivre-Finn C, Hendriks LE, et al. Small-cell lung cancer: ESMO Clinical Practice Guidelines for diagnosis, treatment and follow-up. Ann Oncol. 2021;32(7):839–53. 10.1016/j.annonc.2021.03.207 33864941 PMC9464246

[2] Turrisi AT, 3rd, Kim K, Blum R, Sause WT, Livingston RB, Komaki R, et al. Twice-daily compared with once-daily thoracic radiotherapy in limited small-cell lung cancer treated concurrently with cisplatin and etoposide. N Engl J Med. 1999;340(4):265–71. 10.1056/NEJM199901283400403 9920950

[3] Higgins KA, Gorgens S, Sudmeier LJ, Faivre-Finn C. Recent developments in limited stage small cell lung cancer. Transl Lung Cancer Res. 2019;8(Suppl 2):S147–52. 10.21037/tlcr.2019.05.13 31673519 PMC6795581

[4] Faivre-Finn C, Snee M, Ashcroft L, Appel W, Barlesi F, Bhatnagar A, et al. Concurrent once-daily versus twice-daily chemoradiotherapy in patients with limited-stage small-cell lung cancer (CONVERT): an open-label, phase 3, randomised, superiority trial. Lancet Oncol. 2017;18(8):1116–25. 10.1016/S1470-2045(17)30318-2 28642008 PMC5555437

[5] Gronberg BH, Killingberg KT, Flotten O, Brustugun OT, Hornslien K, Madebo T, et al. High-dose versus standard-dose twice-daily thoracic radiotherapy for patients with limited stage small-cell lung cancer: an open-label, randomised, phase 2 trial. Lancet Oncol. 2021;22(3):321–31. 10.1016/S1470-2045(20)30742-7 33662285

[6] Winther-Larsen A, Hoffmann L, Moeller DS, Khalil AA, Knap MM. Evaluation of factors associated with loco-regional failure and survival in limited disease small cell lung cancer patients treated with chemoradiotherapy. Acta Oncol. 2015;54(9):1574–81. 10.3109/0284186X.2015.1062135 26203924

[7] Seute T, Leffers P, ten Velde GP, Twijnstra A. Neurologic disorders in 432 consecutive patients with small cell lung carcinoma. Cancer. 2004;100(4):801–6. 10.1002/cncr.20043 14770437

[8] Auperin A, Arriagada R, Pignon JP, Le Pechoux C, Gregor A, Stephens RJ, et al. Prophylactic cranial irradiation for patients with small-cell lung cancer in complete remission. Prophylactic Cranial Irradiation Overview Collaborative Group. N Engl J Med. 1999;341(7):476–84. 10.1056/NEJM199908123410703 10441603

[9] Slotman B, Faivre-Finn C, Kramer G, Rankin E, Snee M, Hatton M, et al. Prophylactic cranial irradiation in extensive small-cell lung cancer. N Engl J Med. 2007;357(7):664–72. 10.1056/NEJMoa071780 17699816

[10] Hochstenbag MM, Twijnstra A, Wilmink JT, Wouters EF, ten Velde GP. Asymptomatic brain metastases (BM) in small cell lung cancer (SCLC): MR-imaging is useful at initial diagnosis. J Neurooncol. 2000;48(3):243–8. 10.1023/a:1006427407281 11100822

[11] Seute T, Leffers P, ten Velde GP, Twijnstra A. Detection of brain metastases from small cell lung cancer: consequences of changing imaging techniques (CT versus MRI). Cancer. 2008;112(8):1827–34. 10.1002/cncr.23361 18311784

[12] Gaebe K, Erickson AW, Li AY, Youssef AN, Sharma B, Chan KKW, et al. Re-examining prophylactic cranial irradiation in small cell lung cancer: a systematic review and meta-analysis. EClinicalMedicine. 2024;67:102396. 10.1016/j.eclinm.2023.102396 38261885 PMC10796984

[13] Takahashi T, Yamanaka T, Seto T, Harada H, Nokihara H, Saka H, et al. Prophylactic cranial irradiation versus observation in patients with extensive-disease small-cell lung cancer: a multicentre, randomised, open-label, phase 3 trial. Lancet Oncol. 2017;18(5):663–71. 10.1016/S1470-2045(17)30230-9 28343976

[14] Wolfson AH, Bae K, Komaki R, Meyers C, Movsas B, Le Pechoux C, et al. Primary analysis of a phase II randomized trial Radiation Therapy Oncology Group (RTOG) 0212: impact of different total doses and schedules of prophylactic cranial irradiation on chronic neurotoxicity and quality of life for patients with limited-disease small-cell lung cancer. Int J Radiat Oncol Biol Phys. 2011;81(1):77–84. 10.1016/j.ijrobp.2010.05.013 20800380 PMC3024447

[15] Belderbos JSA, De Ruysscher DKM, De Jaeger K, Koppe F, Lambrecht MLF, Lievens YN, et al. Phase 3 randomized trial of prophylactic cranial irradiation with or without hippocampus avoidance in SCLC (NCT01780675). J Thorac Oncol. 2021;16(5):840–9. 10.1016/j.jtho.2020.12.024 33545387

[16] de Ruiter MB, Groot PFC, Deprez S, Pullens P, Sunaert S, de Ruysscher D, et al. Hippocampal avoidance prophylactic cranial irradiation (HA-PCI) for small cell lung cancer reduces hippocampal atrophy compared to conventional PCI. Neuro Oncol. 2023;25(1):167–76. 10.1093/neuonc/noac148 35640975 PMC9825336

[17] Rodriguez de Dios N, Counago F, Murcia-Mejia M, Rico-Oses M, Calvo-Crespo P, Samper P, et al. Randomized phase III trial of prophylactic cranial irradiation with or without hippocampal avoidance for small-cell lung cancer (PREMER): a GICOR-GOECP-SEOR study. J Clin Oncol. 2021;39(28):3118–27. 10.1200/JCO.21.00639 34379442

[18] Daly ME, Ismaila N, Decker RH, Higgins K, Owen D, Saxena A, et al. Radiation therapy for small-cell lung cancer: ASCO guideline endorsement of an ASTRO guideline. J Clin Oncol. 2021;39(8):931–9. 10.1200/JCO.20.03364 33502911

[19] Qiu B, Li Q, Liu J, Huang Y, Pang Q, Zhu Z, et al. Moderately hypofractionated once-daily compared with twice-daily thoracic radiation therapy concurrently with etoposide and cisplatin in limited-stage small cell lung cancer: a multicenter, Phase II, randomized trial. Int J Radiat Oncol Biol Phys. 2021;111(2):424–35. 10.1016/j.ijrobp.2021.05.003 33992717

[20] Cheng Y, Spigel DR, Cho BC, Laktionov KK, Fang J, Chen Y, et al. Durvalumab after chemoradiotherapy in limited-stage small-cell lung cancer. N Engl J Med. 2024;391(14):1313–27. 10.1056/NEJMoa2404873 39268857

[21] Møller DS, Holt MI, Alber M, Tvilum M, Khalil AA, Knap MM, et al. Adaptive radiotherapy for advanced lung cancer ensures target coverage and decreases lung dose. Radiother Oncol. 2016;121(1):32–8. 10.1016/j.radonc.2016.08.019 27647459

[22] Wahlund LO, Barkhof F, Fazekas F, Bronge L, Augustin M, Sjögren M, et al. A new rating scale for age-related white matter changes applicable to MRI and CT. Stroke. 2001;32(6):1318–22. 10.1161/01.STR.32.6.1318 11387493

[23] Longstreth WT, Jr, Manolio TA, Arnold A, Burke GL, Bryan N, Jungreis CA, et al. Clinical correlates of white matter findings on cranial magnetic resonance imaging of 3301 elderly people. The Cardiovascular Health Study. Stroke. 1996;27(8):1274–82. 10.1161/01.str.27.8.1274 8711786

[24] Andreatta Maduro P, Guimarães MP, de Sousa Rodrigues M, Pereira Rolim Coimbra Pinto AP, da Mota Junior AA, Lima Rocha AS, et al. Comparing the efficacy of two cognitive screening tools in identifying gray and white matter brain damage among older adults. J Aging Res. 2024;2024:5527225. 10.1155/2024/5527225 38690079 PMC11060871

[25] Noh T, Walbert T. Brain metastasis: clinical manifestations, symptom management, and palliative care. Handb Clin Neurol. 2018;149:75–88. 10.1016/B978-0-12-811161-1.00006-2 29307363

[26] Jena A, Taneja S, Talwar V, Sharma JB. Magnetic resonance (MR) patterns of brain metastasis in lung cancer patients: correlation of imaging findings with symptom. J Thorac Oncol. 2008;3(2):140–4. 10.1097/JTO.0b013e318161d775 18303434

[27] Horn L, Mansfield AS, Szczęsna A, Havel L, Krzakowski M, Hochmair MJ, et al. First-line atezolizumab plus chemotherapy in extensive-stage small-cell lung cancer. N Engl J Med. 2018;379(23):2220–9. 10.1056/NEJMoa1809064 30280641

[28] Paz-Ares L, Dvorkin M, Chen Y, Reinmuth N, Hotta K, Trukhin D, et al. Durvalumab plus platinum-etoposide versus platinum-etoposide in first-line treatment of extensive-stage small-cell lung cancer (CASPIAN): a randomised, controlled, open-label, phase 3 trial. Lancet. 2019;394(10212):1929–39. 10.1016/s0140-6736(19)32222-6 31590988

[29] Varlotto J, Voland R, DeCamp M, Khatri J, Shweihat Y, Nwanwene K, et al. Role of consolidative thoracic and prophylactic cranial radiation in extensive stage small cell lung cancer in chemo-immunotherapy era. Radiother Oncol. 2025;202. 10.1016/j.radonc.2024.110619 39537032

