# Supplementary material for: Role of prophylactic cranial irradiation in patients with limited disease small cell lung cancer: A Danish single institution cohort
Source: Acta Oncol. 2025 Jul 25;64:43935. doi: 10.2340/1651-226X.2025.43935 (PMC12308536; doi:10.2340/1651-226X.2025.43935)
Supplement: Supplementary file 1 [file AO-64-43935-s1.pdf]

Supplementary material has been published as submitted. It has not been copyedited, or typeset by Acta Oncologica

# Supplementary material

**Supplementary Table** for table 2 of the article. Results of the univariate and multivariate Cox Regression for age.

| | **Univariate Cox Regression** | | **Multivariate Cox Regression*** | |
|---|---|---|---|---|
| | **HR (95% CI)** | **p** | **HR (95% CI)** | **p** |
| **Age** | 1.00 (0.99-1.02) | 0.68 | 1.00 (0.98-1.02) | 0.75 |

*If including age, sex, PS, stage, chemotherapy agent, treatment period and PCI.

**Supplementary Figure 1** Forest Plot of the hazard ratio for death from the multivariate Cox Regression including sex, PS, stage, chemotherapy agent, treatment period and PCI.

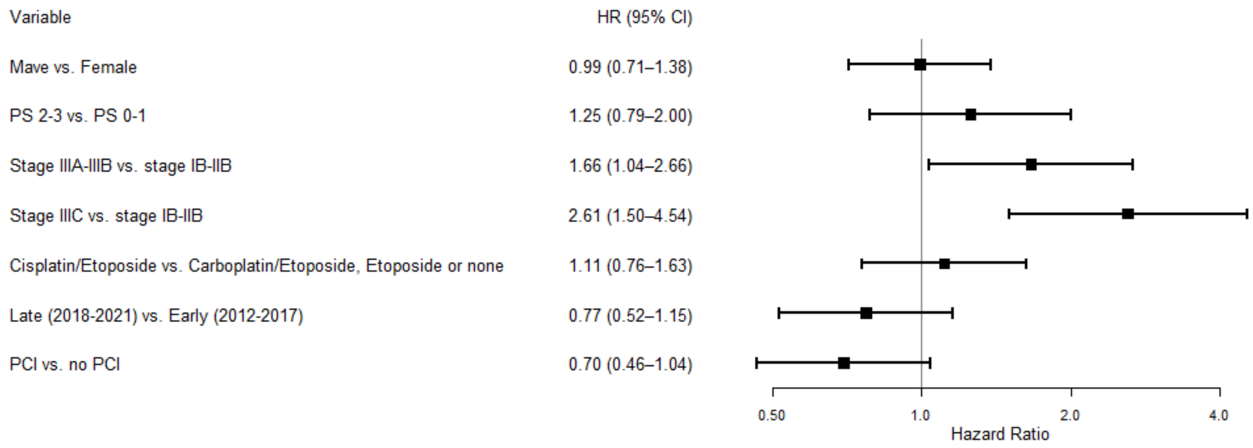


**Supplementary Figure 2**

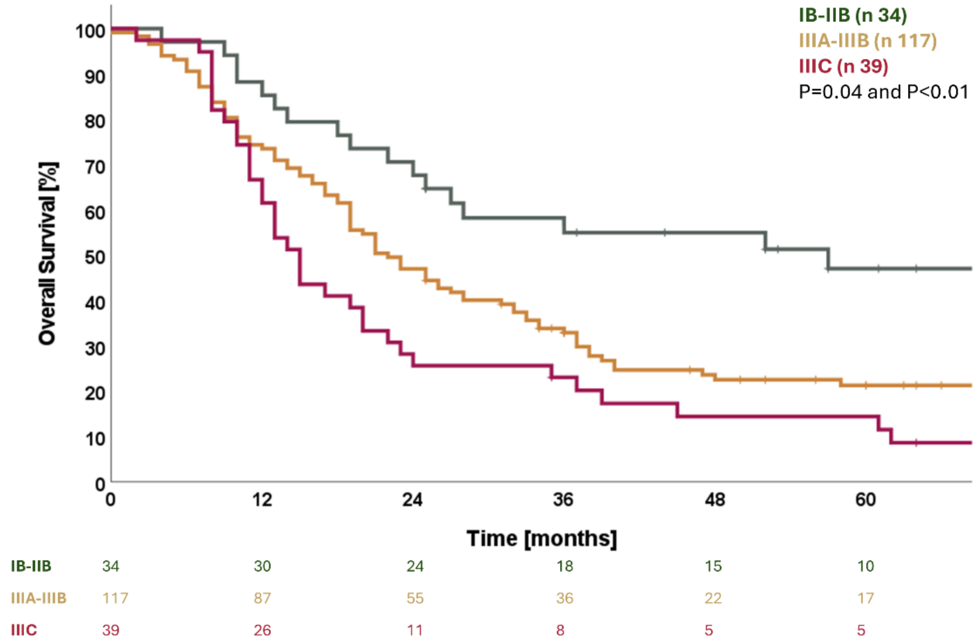


Kaplan Meier curves of Overall Survival in all 190 patients in the cohort. Compared in groups by univariate Cox Regression, stage IIIA-IIIC (n=117) vs. IB-IIB (n=34) p=0.04 and stage IIIC (n=39) vs. IB-IIB (n=34) p<0.01

**Supplementary Figure 3**

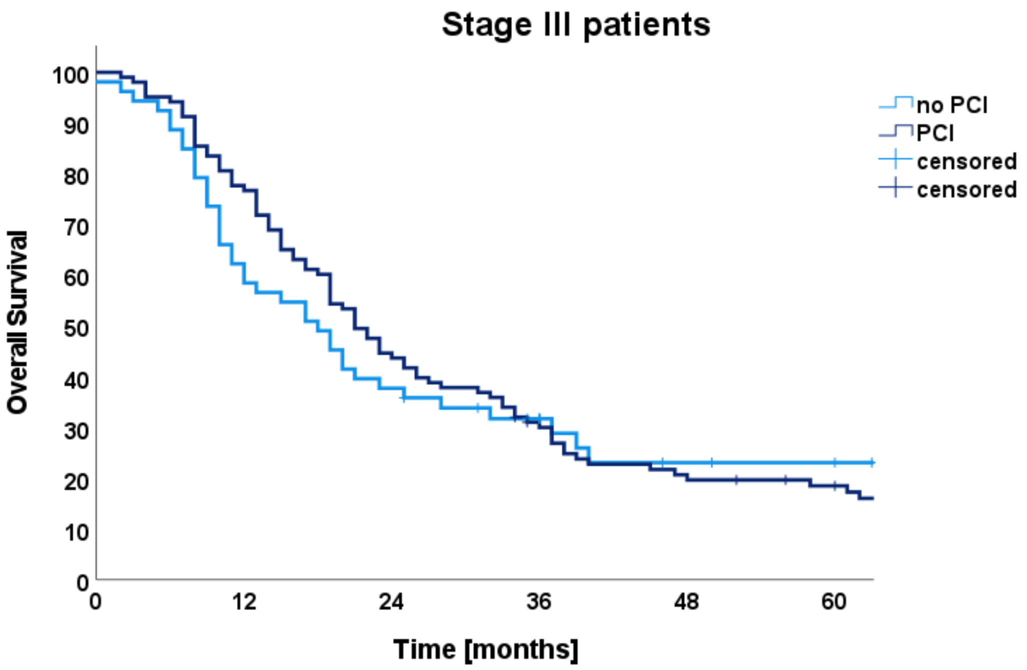


Kaplan Meier curves for Overall Survival [%] in156 patients in the cohort with stage III disease, no PCI (n=53) vs. PCI (n=103), compared by univariate Cox Regression p=0.67

**Supplementary Figure 4**

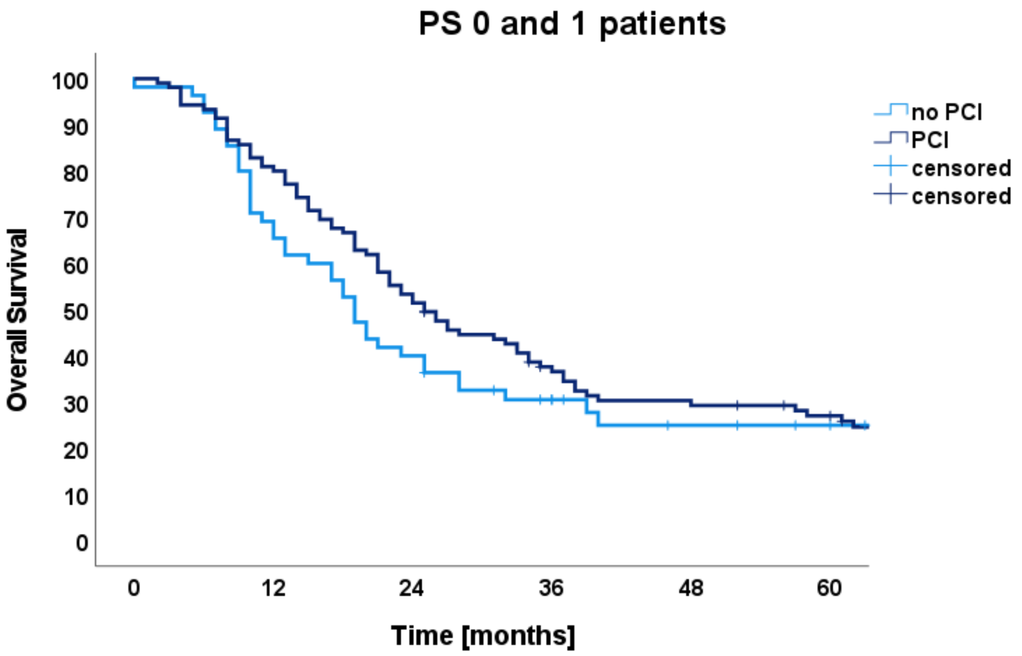


Kaplan Meier curves for Overall Survival [%] in 160 patients in the cohort with PS 0 or 1, no PCI (n=55) vs. PCI (n=105), compared by univariate Cox Regression p=0.22
